# Carriage of the JP2 Genotype of *Aggregatibacter actinomycetemcomitans* by Periodontitis Patients of Various Geographic Origin, Living in Sweden

**DOI:** 10.3390/pathogens11111233

**Published:** 2022-10-25

**Authors:** Rolf Claesson, Jan Oscarsson, Anders Johansson

**Affiliations:** Department of Odontology, Umeå University, SE-901 87 Umeå, Sweden

**Keywords:** *Aggregatibacter actinomycetemcomitans*, JP2 genotype, periodontitis, Sweden, adolescents

## Abstract

The JP2 genotype of *Aggregatibacter actinomycetemcomitans* serotype b is associated with aggressive forms of periodontitis and was initially identified as affecting adolescents in North and West Africa. The dissemination of this genotype follows the migration routes and can today be detected in samples from periodontitis patients in a high number of countries. In the present study, we aim to describe findings of the JP2 genotype *A. actinomycetemcomits* in a clinical laboratory at the Dental School, Odontology, Umeå University, Sweden. The findings of JP2 carriers are documented during a 21-year period, and the age and geographic origin of the sampled individuals are described. In addition, the collected JP2 isolates were separated into North or West African origin by analyses of the presence of a point mutation in the *hbpA2* pseudogene of the bacterium. In a total of 2296 sampled individuals during this period in this Swedish population of periodontitis patients, 32 JP2 carriers were detected by cultivation and PCR. The geographic background of these individuals was diverse, including sixteen with African origin, ten with a Swedish origin and six additional ones with a non-African origin. The JP2 genotypes of *A. actinomycetemcomitans* were mainly isolated from young individuals (<35 years of age), and seven out of the 32 isolates were of a West African origin based on the sequence of *hbpA2*. We conclude that the JP2 genotype of *A. actinomycetemcomitans* can be detected world-wide in subgingival plaque samples from adolescents affected by periodontitis.

## 1. Introduction

The Gram-negative bacterial species *Aggregatibacter actinomycetemcomitans* is associated with aggressive forms of periodontitis in young individuals [1,2]. The most studied virulence factor of this bacterium is the leukotoxin (LtxA), a protein which induces the release of the osteoclast-activating cytokine interleukin (IL)-1β from human macrophages and also kills neutrophils [3,4]. *A. actinomycetemcomitans* is distributed into seven serotypes (a–g) [5]. A specific variant of the serotype b, the JP2 genotype, produces large amounts of leukotoxin and is characterized by the absence of a 530-base pair (bp) sequence within the *ltxCABD* promoter region [6,7].

The origin of the JP2 genotype was initially suggested to be among individuals living in the Mediterranean part of Africa, and then later on, following a dissemination route through West Africa, and further to North and South America via the transatlantic slave trade [8,9]. Today, there are several reports on the carriage of the JP2 genotype by individuals outside the North and West African regions [10,11,12,13,14,15,16,17,18,19]. 

Carriers of the JP2 genotype of *A. actinomycetemcomitans* are at a highly increased risk of being affected by an aggressive form of periodontitis, which affects young individuals [20,21]. Thus, it is important to early identify individuals colonized with this genotype. In light of the general dental health, it is important to also monitor the global spreading of the JP2 genotype. We have earlier reported on findings of the JP2 genotype of *A. actinomycetemcomitans* colonizing periodontitis patients living in Sweden [11,22]. The aim of this work is to summarize findings regarding this genotype in samples from Swedish inhabitants with periodontitis analyzed during a 21-year period at the clinical laboratory, Dental school, Umeå, Sweden. We also discuss important factors such as age, geographic origin, and family relations with regards to the detection of *A. actinomycetemcomitans* and its JP2 genotype.

## 2. Materials and Methods

For clinical laboratory diagnostics and treatment purposes, subgingival samples have been analyzed at the clinical laboratory of the Dental School, Odontology, Umeå University, Sweden, for more than 30 years [23]. In addition to the determination of the proportion of *A. actinomycetemcomitans* (% of total viable count (TVC)) in the samples, the isolates have been further characterized as described in brief below. This study is a summary of data obtained from clinical samples sent to the clinical laboratory for identification and characterization. No sample has been taken for research purposes, and the data cannot be traced to any of the sampled individuals. Selected results including microbiological findings from all subgingival plaque samples that have been sent to the clinical laboratory for clinical diagnostic purpose during the years 2000 to 2020 are included in the study, but no clinical characteristics regarding the patients have been included. The information is based on 5162 samples from 2296 periodontitis patients and patient origin provided by the clinician.

### 2.1. Detection and Cultivation of A. actinomycetemcomitans

Cultivation of the samples was performed as described in detail by Claesson et al. [11]. Briefly, after transportation of the samples in VMGAIII-medium, and serial dilution, the samples were spread on blood agar plate, and, in addition, on an *A. actinomycetemcomitans* selective medium, trypticase-bacitracin-vancomycin (TBV) [24,25]. After determining the TVC and numbers of *A. actinomycetemcomitans* colonies on the blood agar and TBV plates, respectively, the percentage of *A. actinomycetemcomitans* in the samples was calculated. 

### 2.2. Genetic Characterization of A. actinomycetemcomitans

Leukotoxin promoter typing and determination if carriers of the bacterium had the variant originating from West Africa or from the Mediterranean area were performed by DNA-based methods. The latter characterization was based on one single nucleotide polymorphism (SNP) in the *hbpA2* pseudogene. All oligonucleotide primers and PCR cycling conditions used for these characterizations of *A. actinomycetemcomitans* isolates collected from the TBV-plates cultivated from samples from the patients are described in the works by Haubek et al., and Höglund Åberg et al., respectively [8,26].

### 2.3. Statistical Analyses

Statistical significance has been performed using the Mann–Whitney U-test (SPSS Inc., Chicago, IL, USA). 

## 3. Results

### 3.1. Prevalence and Age of the JP2 Genotype Carriers

**Figure 1:**
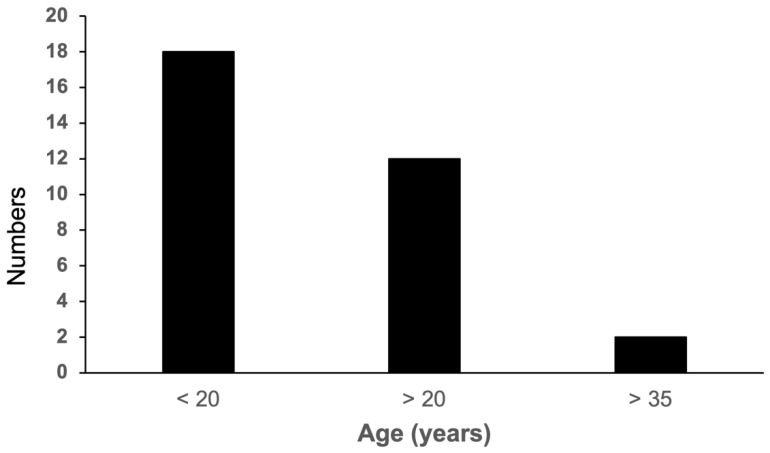
The age of the periodontitis patients carrying the JP2 genotype distributed in three groups.

When the results from 2296 periodontitis patients sampled during 2000–2020 were summarized, 32 *A. actinomycetemcomitans* JP2 genotype carriers were identified, yielding a prevalence of 1.4% regarding carriage (Table 1). Moreover, as 30 of the JP2 carriers were distributed among 674 young (<35 years of age) patients, the prevalence of the JP2 genotype among these individuals reached 4.4% (Figure 1). 

As determined in the present study, 18 out of 32 JP2 genotype carriers were in fact below 20 years of age, and only two were above 35 (Table 1).

### 3.2. Geographic Origin of the JP2 Genotype Carriers

**Figure 2:**
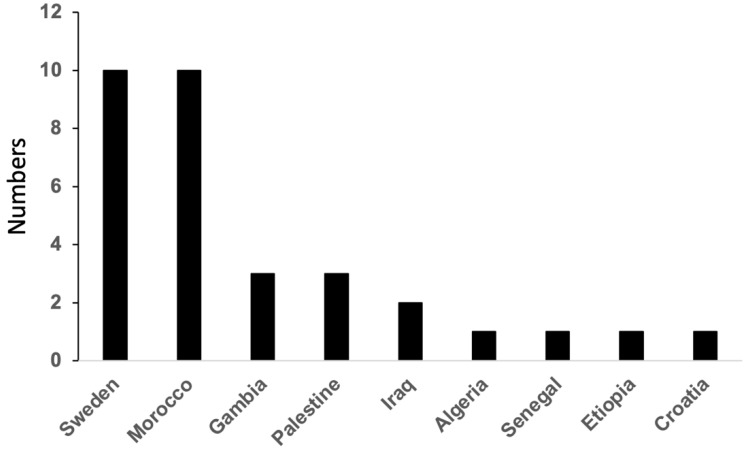
Distribution of JP2 genotype carriers in relation to their geographic origin.

Among the 32 periodontitis patients carrying a JP2 genotype of *A. actinomycetemcomitans*, sixteen were of African origin, ten of Swedish origin, and six additional ones were also of non-African, albeit non-Swedish origin (Figure 2). This further supports earlier reports that JP2 carriers are not exclusively of African origin [13]. The West African variant of the *A. actinomycetemcomitans* JP2 genotype is characterized by a specific SNP within the *hbpA2* pseudogene [8]. This characteristic is an important tool for studying the worldwide dissemination of the JP2 genotype. As deduced from the present study, four of the 16 JP2 isolates with African origin could be traced as West African (W) and two of the 16 JP2 isolates with non-African origin, separated by an SNP on the *hbpA2* pseudogene (Table 1).

### 3.3. Proportions of JP2 Genotype A. actinomycetemcomitans in Plaque Samples

In this study, 5065 samples were collected from 2264 JP2-negative patients and 97 samples from 32 JP2 patients. When the samples were distributed in groups with regards to genotype (JP2/non-JP2) and proportions (%) of *A. actinomycetemcomitans* of TVC in the samples, it became apparent that the JP2 variant of the bacterium had a higher capacity to colonize the periodontal pocket than the non-JP2 variant (*p* < 0.001) (Table 2).

## 4. Discussion

In the present study, we report on the findings of *A. actinomycetemcomitans* and its JP2 genotype during a time-period from 2000 to 2020 in the clinical laboratory at the dental school in Umeå, Sweden [23]. Based on the data collected in the present study, the JP2 genotype was detected in 1.4% (32) of the sampled individuals. The study is based on observations from the clinical laboratory without access to any clinical parameters. This is a limitation of the study and prevents correlations to clinical observations; however, patients analyzed for the carriage of pathogenic bacterial species are associated with a history of periodontal disease [27]. *A. actinomycetemcomitans* is the bacterial species to have in focus in this context, especially its JP2 genotype, and possibly also the *cagE* genotype, to which all hitherto known JP2 genotype strains belong [28,29]. During our work to analyze periodontal pocket samples for the presence of *A. actinomycetemcomitans*, we have identified three patients carrying leukotoxin promoter types other than JP2. These findings, which are further described in the works by Claesson et al. [30,31], show that the present procedures for leukotoxin promoter typing are also usable beyond the JP2 genotype.

An important factor to have in mind is that aggressive forms of periodontitis are initiated early in life [32,33]. Thirty of the 32 JP-positive individuals that were detected in the present study were below 35 years of age. This further supports earlier observations that the carriage of the JP2 genotype is age related [11,34]. In turn, this increases the risk that young carriers of the JP2 genotype are overlooked and are at risk to be affected by an aggressive form of periodontal disease. The absence of the JP2 genotype in periodontal pockets of older patients (>35 years of age) does not exclude the fact that a JP2-associated onset of periodontal disease has occurred at younger ages. It is possible that this genotype have been present in an early phase of disease but was eradicated from the site of infection due to competition from other bacterial species and/or to other unknown factors counteracting survival/growth of the JP2 genotype. Among the 32 patients carrying a JP2 genotype assessed in the present work, siblings have been found to carry the JP2 genotype in three cases. This type of vertical spreading of the bacterium should also be taken into consideration [35,36]. 

Taken together, the global prevalence of the JP2 genotype is rather low in the general population. However, a high prevalence of this genotype has been reported in adolescents in Morocco (10%) [21] and Ghana (8.8%) [37]. Thus, for studying the dissemination of this genotype, it would be beneficial to focus on patients suffering from an aggressive form of periodontal disease. 

## 5. Conclusions

Our take-home message with the present study is that the JP2 genotype of *A. actinomycetemcomitans* can be found outside Africa and among people with an origin other than African. This could be important information for clinicians and patients when periodontitis unexpectedly affects otherwise healthy young individuals with proper oral hygiene.

## Figures and Tables

**Table 1:** The 32 *A. actinomycetemcomitans* JP2 genotype isolates assessed in the present study. All were collected from periodontitis patients residing in Sweden and were characterized at the clinical laboratory, Dental School, Odontology, Umeå, Sweden. M/W indicates “Mediterranean” and “West African” origin of the JP2 isolate, respectively, as deduced by the sequence of *hbpA2*.

Number	Name of Isolate	Year of Isolation	Origin of Patient	Age	M/W
1	133A1	2008	Sweden	33	M
2	BL1	2008	Sweden	63	M
3	520A	2001	Sweden	20	W
4	246A1	2004	Algeria	43	M
5	090A	2010	Sweden	27	M
6	196A1	2010	Croatia	23	M
7	115A1	2011	Iraq	18	M
8	352B	2011	Iraq	23	M
9	245	2011	Sweden	18	M
10	557A1	2012	Gambia	19	W
11	338A1	2013	Sweden	31	M
12	342A1	2013	Morocco	15	M
13	408A1	2013	Gambia	15	W
14	304A1	2014	Sweden	15	M
15	361A1	2014	Morocco	15	M
16	698A1	2014	Morocco	16	M
17	012A1	2015	Morocco	16	M
18	299A1	2015	Sweden	23	W
19	087B1	2016	Morocco	14	M
20	096A1	2016	Morocco	17	M
21	315A1	2016	Palestine	18	M
22	741A1	2016	Sverige	34	M
23	311A1	2019	Gambia	23	W
24	419A1	2019	Palestine	18	M
25	357A1	2019	Ethiopia	30	W
26	585A1	2019	Morocco	20	M
27	074	2020	Sweden	16	M
28	156	2020	Palestine	17	M
29	170	2020	Senegal	31	W
30	172	2020	Morocco	14	M
31	199A1	2020	Morocco	22	M
32	290A2	2020	Morocco	13	M

**Table 2:** Distribution of *A. actinomycetemcomitans* groups with regard to genotype, proportions (%) of this species of the total viable count, and numbers (within brackets) of analyzed samples.

JP2/Non-JP2 Carriers	0%	0.1–1%	>1–5%	>5–25%	>25–50%	>50%	Total Numbers
non-JP2	78.6 (3981)	5.9 (301)	4.7 (237)	5.5 (276)	2.0 (103)	3.3 (167)	5065
JP2	32.0 (31)	11.3 (11)	9.3 (9)	12.4 (12)	8.2 (8)	26.8 (26)	97

0%: samples without detection of *A. actinomycetemcomitans*.

## Data Availability

Data is available from the corresponding author.
